# Latent Profiles in the Perception of Knowledge in Emotional Education During Initial Teacher Training: A Retrospective Study of Chilean In-Service Teachers

**DOI:** 10.3390/bs16091469

**Published:** 2026-08-24

**Authors:** Gerardo Fuentes-Vilugrón, Francisco Correa-Araneda, Carlos Arriagada-Hernández, Felipe Caamaño-Navarrete, Lorena Jara-Tomckowiack

**Affiliations:** 1 Facultad de Educación, Universidad Católica de Temuco, Temuco 4780000, Chile; gerardo.fuentes@uct.cl (G.F.-V.); lorena.jarat@fmda.cl (L.J.-T.); 2 Facultad de Salud, Universidad Santo Tomás, Temuco 4780000, Chile; 3 Centro de Gestión y Tecnologías del Agua, Facultad de Ingeniería y Ciencias, Universidad de La Frontera, Temuco 4811230, Chile; 4 Facultad de Educación, Universidad Autónoma de Chile, Temuco 4780000, Chile; carlos.arriagada@uautonoma.cl (C.A.-H.); felipe.caamano@uautonoma.cl (F.C.-N.)

**Keywords:** emotional education, mood, emotional regulation, psychosocial well-being, identity, latent profiles

## Abstract

Emotional education is fundamental in teacher training due to its impact on teacher well-being and student learning. In Chile, initial teacher training has addressed this dimension in a fragmented manner, and little is known about whether in-service teachers exhibit distinct patterns of retrospectively perceived preparation across different domains of emotional education. This study aimed to identify and characterize latent profiles of retrospectively perceived knowledge in emotional education acquired during initial teacher training among Chilean in-service teachers. Using a retrospective design, 412 practicing teachers participated (86.7% female; mean age = 39.2 years; mean teaching experience = 12.6 years). The EEITT Scale assessed perceived knowledge acquired in Mood, Emotional Regulation, Psychosocial Well-being, and Identity. Confirmatory factor analysis supported the predefined four-factor structure, and latent profile analysis was conducted in R by comparing solutions from one to eight profiles. Six profiles were retained, showing a combination of differences in overall perceived preparation and more specific configurations across dimensions. Emotional Regulation emerged as one of the most discriminating dimensions, particularly in profiles characterized by comparatively low perceived preparation in this domain, alone or together with Psychosocial Well-being. The six-profile solution showed adequate classification quality (entropy = 0.824), although classification uncertainty was greater for one intermediate profile. Overall, the findings indicate that teachers do not report a uniform pattern of perceived preparation in emotional education; instead, distinct subgroups differ in both the overall level and configuration of perceived preparation across domains. These results suggest that reliance on sample-level averages may obscure meaningful variation in teachers’ retrospective perceptions of their initial training and may inform future research on differentiated professional development strategies.

## 1. Introduction

In recent years, the study of emotions has gained growing recognition in human development and in teaching and learning processes. In this regard, emotion and learning are interconnected dimensions of the educational experience (G. A. Fuentes-Vilugrón & Riquelme-Mella, 2023; Ibáñez, 2002; Riquelme et al., 2019). Historically, learning has been mediated by pedagogical practices and the ways in which students regulate their emotions. From a neuroscientific perspective, it has been shown that emotional and cognitive processes are inextricably linked in the brain, sharing structures such as the amygdala and the prefrontal cortex, which explains why meaningful learning cannot occur without adequate emotional processing (Immordino-Yang & Damasio, 2007; Miño-Díaz et al., 2025; Panksepp, 2011). However, the traditional educational perspective kept affective life outside the school classroom by prioritizing rationality as the core of educational processes (De Sousa, 1990). This dichotomy between reason and emotion, inherited from the Western philosophical tradition dating back to Plato and Descartes, has permeated school practices for centuries, relegating emotions to the realm of the private or the “irrational” (Immordino-Yang & Damasio, 2007).

In this sense, those educational actors (families and teachers) who act as shapers of emotional regulation through socialization mechanisms grounded in the adults’ own beliefs and life experiences are of great relevance (DeLoretta, 2024; Denham, 2023; Edler & Valentino, 2024). The emotional socialization theory of Eisenberg et al. (1998) posits that adults transmit to children not only explicit regulation strategies but also implicit values about which emotions are acceptable, how they should be expressed, and under what circumstances. This process occurs both at home and at school, shaping the ‘emotional repertoire’ that children will display throughout their lives (Denham et al., 2015). This repertoire encompasses multiple dimensions of emotional functioning that are relevant to educational contexts. These processes may contribute to how children and young people understand, express, and regulate their emotions and behavior.

In this context, teachers, as one of the main educational actors, assume a fundamental role in emotional education, due to their participation as primary agents of emotional socialization, influencing students through three interrelated dimensions: (a) modeling: where teachers, through their own emotional behavior, offer a model of regulation that students observe and internalize (Bandura, 1977); (b) reactions to emotions: Teachers’ responses to students’ emotional expressions convey which emotions are acceptable, how they should be expressed, and under what circumstances (Denham et al., 2015; Eisenberg et al., 1998); and (c) explicit teaching: Through planned activities, intentional conversations, and reflective spaces, teachers can deliberately teach emotional regulation strategies (Brackett et al., 2025; Durlak et al., 2011; Hoffmann et al., 2020). However, previous research has stated that teachers in Chile do not have specific and systematic academic training to assume these functions (G. Fuentes-Vilugrón et al., 2023). This situation is not unique to Chile; international evidence suggests that emotional training in initial teacher preparation remains fragmented across various educational systems, with Spain, the United States, and Finland showing heterogeneous approaches that nonetheless share the challenge of integrating emotional competencies into curricular structures (Bisquerra, 2016; Brackett et al., 2025; Corcoran & Tormey, 2013).

These challenges may have implications for teacher well-being and professional practice, particularly in contexts characterized by burnout, mental health difficulties, and demands on executive functions such as emotional regulation, decision-making, and working memory, all of which are relevant to effective teaching (Bayes et al., 2021; G. Fuentes-Vilugrón et al., 2025a; Koutsimani et al., 2019). However, teachers have often preferred to neglect and/or suppress emotions due to the belief that these could become a threat to the objectives set by the school system (M.-L. Chang, 2020; V. T. Chang et al., 2018). This phenomenon, known as “emotional suppression” is particularly relevant to the present study because it highlights the potential implications of limited perceived preparation in emotional education for how teachers understand and respond to emotional demands in professional contexts (Grandey, 2000; Hülsheger et al., 2013). For this reason, emotional education cannot be studied simplistically as a set of techniques but rather as a complex process contextualized to the subjective and professional reality of those who are part of and responsible for the teaching processes.

Initial teacher training (ITT) is a period in which future teachers acquire a set of knowledge, skills, and attitudes intended to support their subsequent professional practice. This training period, which in Chile lasts an average of 8 to 10 semesters depending on the program, constitutes the critical window for the development of professional competencies that will support teaching practice (Ávalos, 2014). As highlighted in the previous section, emotional education has presented significant challenges and needs that have affected teaching practice and, consequently, teaching and learning processes. In the Chilean context, modifications to ITT have been developed over the last decade, including through the publication of Law 20.903 on Teacher Professional Development (Biblioteca del Congreso Nacional [BCN], 2016), creating the Teacher Professional Development System which includes: (a) Teaching Profession Standards; (b) National Diagnostic Assessment; and (c) Mandatory Accreditation of Pedagogy Programs. This law, driven by the 2011 social movement that placed the quality of teacher training at the center of the public agenda, represented a milestone in the regulation of pedagogy programs, establishing for the first time mandatory standards and quality assurance mechanisms (Biblioteca del Congreso Nacional [BCN], 2016).

However, the emotional dimension has not gained relevance in the initiatives developed so far, revealing persistent limitations in the systematic incorporation of emotional education into initial teacher training (Bächler Silva et al., 2020). For example, the initial teacher training standards emphasize disciplinary knowledge, management of strategies, and classroom management, leaving aside aspects related to competencies for educating emotions and for teacher emotional regulation. This reveals a process of omission of this dimension in Chilean training processes (Riquelme et al., 2019), highlighting the gap between the emotional competencies required in the classroom and those developed during ITT (G. Fuentes-Vilugrón et al., 2025b; Jennings & Greenberg, 2009). Consequently, teachers may enter the school system perceiving limitations in their preparation to address the emotional demands of the profession, a concern that is especially relevant during the early years of professional practice (Orrego-Tapia, 2023). Person-centered approaches complement variable-centered analyses by examining whether individuals exhibit distinct configurations across multiple dimensions rather than assuming a homogeneous population (Bergman & Trost, 2006; Spurk et al., 2020). In the present context, this approach is particularly relevant because different dimensions of retrospectively perceived preparation may combine in patterns that are not apparent from sample-level averages or associations between variables. LPA therefore provides an appropriate framework for identifying heterogeneity in how teachers retrospectively perceive their preparation in emotional education.

Therefore, the study aims to identify and characterize latent profiles of retrospectively perceived knowledge in emotional education acquired during initial teacher training in a sample of practicing Chilean teachers. To address this aim, the study examines the following research questions (RQ):

**RQ1.**  *Does the predefined four-factor structure of the EEITT show adequate fit and internal consistency in the analytical sample of in-service teachers?*

**RQ2.**  *What latent profiles of retrospectively perceived preparation in emotional education can be identified across the dimensions of Mood, Emotional Regulation, Psychosocial Well-being, and Identity?*

**RQ3.**  *How are the identified profiles characterized in terms of their configuration across the four EEITT dimensions and selected sociodemographic characteristics?*

The examination of the EEITT measurement structure serves as a preliminary analytical step supporting the subsequent LPA, whereas profile identification and characterization constitute the central empirical focus of the study. Potential implications for initial teacher education and professional development are considered as directions for future research and practice rather than as intervention outcomes directly evaluated in the present study.

## 2. Materials and Methods

This study used a quantitative methodological approach with latent profile analysis (LPA) to identify latent subgroups of teachers based on their response patterns on the Perceived Knowledge Acquired in Emotional Education during Initial Teacher Training Scale (EEITT). LPA is a finite mixture modeling technique that allows for the identification of latent profiles (unobserved subgroups) based on observed continuous variables (Spurk et al., 2020). A person-centered approach was selected because the study sought to identify heterogeneous configurations of retrospectively perceived preparation across the four EEITT dimensions rather than focusing exclusively on sample-level averages or associations among variables. LPA was therefore appropriate because it allows multiple continuous indicators to be examined simultaneously to identify subgroups with distinct response patterns. The retrospective cross-sectional design was consistent with the aim of examining teachers’ current perceptions of their initial training, without inferring causality or changes over time.

### 2.1. Sample

The sample consisted of 412 practicing Chilean teachers, recruited through non-probability snowball sampling. The inclusion criteria were: (1) being actively teaching in Chile at the time of the study, (2) having completed initial teacher training and having at least one year of teaching experience to ensure an informed retrospective evaluation, and (3) providing informed consent to participate in the research. Participants were recruited through digital platforms and professional teacher networks. Data collection was conducted between April and August 2025. Because recruitment relied on an open snowball strategy and the invitation could be redistributed through professional networks and digital platforms, the total number of individuals who received or viewed the invitation could not be determined; therefore, a conventional response rate could not be calculated. The invitation to participate was disseminated through these channels and included a link to an online questionnaire administered via SurveyMonkey (https://www.surveymonkey.com/). Potential participants received information about the general purpose of the study, the eligibility criteria, and the voluntary nature of participation. Before accessing the questionnaire, participants reviewed the informed consent form and were required to indicate their voluntary agreement to participate. As detailed in Table 1, the sample presented the following sociodemographic characteristics: mean age of 39.2 years (SD = 9.34, range: 23–76), average teaching experience of 12.6 years (SD = 8.52, range: 1–45), and a predominance of females (86.7%). The gender distribution was: 357 females (86.7%), 52 males (12.6%), 1 non-binary person (0.2%), and 2 participants who preferred not to respond (0.5%).

### 2.2. Instrument

To assess the perception of knowledge acquired in emotional education during initial teacher training, the Perceived Knowledge Acquired in Emotional Education during Initial Teacher Training Scale (EEITT), developed by G. Fuentes-Vilugrón et al. (2025b), was used. The scale consists of 23 items distributed across four first-order dimensions (Supplementary Material S1). Each item is answered using a 6-point Likert-type scale ranging from 1 = Strongly disagree to 6 = Strongly agree. Higher scores indicate greater retrospectively perceived knowledge acquired in emotional education during initial teacher training. The four dimensions are:

(a) Mood (6 items, 1–6): Assesses the perception of knowledge received about the importance of positive mood in teaching-learning processes.
(b) Emotional regulation (5 items, 7–11): Measures the perception of knowledge acquired about emotional regulation concepts and tools applicable to the educational context.
(c) Psychosocial well-being (6 items, 12–17): Evaluates the perception of knowledge received about self-concept, self-esteem, and their influence on student well-being.
(d) Identity (6 items, 18–23): Assesses the perception of knowledge acquired about the consideration and development of student identities in the classroom.

### 2.3. Analysis Procedure

Analyses were conducted using R software (version 4.6.0) for descriptive, reliability, correlational, and latent profile analyses. Latent profile models were estimated using the tidyLPA package (version 2.0.2), with mclust (version 6.1.2) as the estimation backend, while confirmatory factor analysis (CFA) was conducted using Jamovi (Version 2.6.45.0). No missing values were present in the 23 EEITT items, the four dimension scores, age, teaching experience, or gender among the 412 participants; therefore, no imputation or casewise deletion was required. The four EEITT dimension scores were recomputed from the item-level responses according to the predefined scoring structure, and their variable assignments were verified before conducting the correlational and latent profile analyses. The analytical strategy was sequential: the predefined four-factor structure of the EEITT was first examined through CFA, specifying four correlated latent factors corresponding to Mood, Emotional Regulation, Psychosocial Well-being, and Identity, and the four dimension scores were subsequently used as continuous indicators in the LPA. The CFA was estimated using maximum likelihood (ML), with the six-point Likert-type items treated as approximately continuous indicators. Model fit was evaluated using the chi-square statistic (χ^2^), Comparative Fit Index (CFI), Tucker–Lewis Index (TLI), Root Mean Square Error of Approximation (RMSEA), and Standardized Root Mean Square Residual (SRMR). Standardized factor loadings were also examined.

The four EEITT dimension scores were subsequently standardized (z-scores) to ensure comparability across dimensions with different variances, a standard practice in latent profile modeling (Spurk et al., 2020). Following the Guidelines for Reporting on Latent Trajectory Studies (GRoLTS; van de Schoot et al., 2017), the latent profile models were specified with class-specific means, equal variances across profiles, and covariances fixed to zero (tidyLPA Model 1). Sequential solutions ranging from one to eight profiles were estimated. The stability of the candidate solutions was additionally examined by repeating the estimation across six different random seeds; the three- through six-profile solutions reproduced identical log-likelihoods, fit indices, entropy values, and class-size configurations across all replications.

The selection of the optimal number of profiles followed an integrative multi-criteria approach, aligned with current methodological recommendations (Nylund-Gibson & Choi, 2018; Spurk et al., 2020). First, sequential models were estimated and compared from a 1-profile solution to an 8-profile solution to assess progressive improvement in fit and to determine whether additional profiles provided substantively meaningful information.

The final decision was based on the joint evaluation of four types of indicators: (1) Parsimonious information criteria: The Bayesian Information Criterion (BIC), the Akaike Information Criterion (AIC), and the sample-size-adjusted Bayesian Information Criterion (SABIC) were considered (Sen & Cohen, 2024; Tein et al., 2013). For each, lower values were preferred, as they penalize model complexity and seek the optimal balance between data fit and parsimony. (2) Classification quality: Entropy was examined, a metric ranging from 0 to 1 that reflects the precision with which the model assigns individuals to profiles. According to established convention (Clark & Muthén, 2009), adequate classification was considered with values above 0.80. Posterior class-membership probabilities were also examined to explicitly assess classification uncertainty. Participants were assigned to their most likely profile according to the highest posterior probability, and average posterior probabilities (AvePP) were calculated for each profile. (3) Comparative statistical significance tests: The Bootstrap Likelihood Ratio Test (BLRT) was used to examine whether a solution with k profiles provided a significantly better fit than the solution with k−1 profiles, using a threshold of *p* < 0.05. (4) Class-size and substantive criteria: A minimum profile size of approximately 5% of the sample was considered as one component of the model-selection process, rather than as a stand-alone exclusion rule (Masyn, 2013), and the solution had to be theoretically interpretable and meaningful in the context of teacher training in emotional education. Model stability and the substantive distinctiveness of the emerging configurations were also considered when selecting the final solution.

The six-profile solution emerged as the most balanced solution when statistical fit, classification quality, class size, parsimony, stability, and substantive interpretability were considered jointly. The six-profile model showed AIC = 3684.71, BIC = 3817.40, and SABIC = 3712.69, with an entropy of 0.824. The BLRT remained statistically significant (*p* = 0.010), supporting improvement over the five-profile solution. All six profiles met the approximately 5% class-size benchmark considered during model selection, representing between 5.34% and 37.14% of the sample. Classification uncertainty was further evaluated using posterior membership probabilities. The overall mean posterior probability for the assigned profile was 0.871, while profile-specific AvePP values ranged from 0.765 to 0.984. Thus, classification quality was generally good at the solution level, although comparatively greater uncertainty was observed for the Average Perceived Preparation profile (AvePP = 0.765).

## 3. Results

The sample consisted of 412 Chilean teachers, predominantly female (86.7%), with a mean age of 39.2 years (SD = 9.34) and an average of 12.6 years of teaching experience (SD = 8.52). As shown in Table 1, scores on the Perceived Knowledge Acquired in Emotional Education during Initial Teacher Training Scale (EEITT) showed an overall mean of 4.43 (SD = 1.07) on a 1-to-6-point scale. The Mood (M = 4.91, SD = 1.05) and Identity (M = 4.86, SD = 1.17) dimensions presented the highest scores, while Emotional Regulation obtained the lowest score (M = 3.54, SD = 1.45).

The reliability analysis of the EEITT, presented in Table 2, revealed good-to-excellent internal consistency. Cronbach’s Alpha coefficient for the total scale was 0.860, with values ranging from 0.893 to 0.917 for the four factors. McDonald’s ω coefficients confirmed this internal consistency, with a value of 0.866 for the total scale and between 0.908 and 0.930 for the individual factors, supporting the reliability of the EEITT scores used in subsequent analyses.

### 3.1. Confirmatory Factor Analysis

A confirmatory factor analysis (CFA) was conducted to examine the predefined four-factor structure of the EEITT Scale in the present sample. The model specified four correlated latent factors corresponding to Mood, Emotional Regulation, Psychosocial Well-being, and Identity. The four-factor model showed an acceptable fit to the data, χ^2^(224) = 792.00, *p* < 0.001, CFI = 0.931, TLI = 0.922, RMSEA = 0.078, 90% CI [0.073, 0.084], and SRMR = 0.045. Standardized factor loadings were statistically significant (*p* < 0.001) and ranged from 0.646 to 0.806 for Mood, 0.759 to 0.867 for Emotional Regulation, 0.784 to 0.933 for Psychosocial Well-being, and 0.792 to 0.874 for Identity. Overall, these results provide support for the predefined four-factor structure of the EEITT in the present sample, supporting the use of the four dimension scores as indicators in the subsequent latent profile analysis.

### 3.2. Latent Profile Analysis

Latent profile analysis identified six distinct configurations of retrospectively perceived knowledge acquired in emotional education during initial teacher training after evaluating models from one to eight profiles and selecting the optimal solution based on statistical, classification, class-size, stability, parsimony, and substantive interpretability criteria (Table 3).

The six-profile solution was retained as the final solution. It showed AIC = 3684.71, BIC = 3817.40, SABIC = 3712.69, and entropy = 0.824. The BLRT was statistically significant (*p* = 0.010), indicating improvement over the five-profile solution. All six profiles met the approximately 5% class-size benchmark considered during model selection, with profile sizes ranging from 5.34% to 37.14% of the sample. In addition, the solution was stable across the six random seeds examined, reproducing identical log-likelihoods, fit indices, entropy values, and class-size configurations.

Although the seven-profile solution yielded slightly lower information criteria (AIC = 3659.77, BIC = 3812.57, SABIC = 3691.98) and a significant BLRT (*p* = 0.010), it generated a profile containing 4.61% of the sample, slightly below the approximately 5% class-size benchmark considered during model selection, and its entropy was slightly lower (0.816). Importantly, the approximately 5% class-size benchmark was not used as a stand-alone exclusion rule. Inspection of the seven-profile solution showed that the main vulnerability configurations were largely preserved, whereas the additional separation primarily reflected finer quantitative subdivisions of patterns already represented in the six-profile solution, without yielding a clearly distinct new cross-dimensional configuration. Therefore, the seven-profile solution provided limited additional substantive information despite its slightly improved information criteria. Increasing the number of profiles from seven to eight was not supported: AIC increased to 3668.60, BIC to 3841.50, and SABIC to 3705.06; entropy decreased substantially to 0.740, and the BLRT was non-significant (*p* = 0.921). Consequently, the six-profile solution was retained as the most parsimonious solution satisfying the combined statistical, classification, class-size, stability, and substantive interpretability criteria.

Classification quality was further examined using posterior class-membership probabilities. The overall mean posterior probability for the assigned profile was 0.871, while profile-specific average posterior probabilities (AvePP) ranged from 0.765 to 0.984. These results indicated generally good classification quality at the solution level, although comparatively greater classification uncertainty was observed for the Average Perceived Preparation profile (AvePP = 0.765).

The most prevalent profile was Consolidated Perceived Preparation (CPP), which included 153 participants (37.1% of the sample). This profile showed consistently high retrospectively perceived knowledge across all four EEITT dimensions and the highest overall mean (M = 5.43).

The second most frequent profile was Average Perceived Preparation (APP), comprising 117 teachers (28.4%). This profile showed scores close to the sample mean across the four standardized EEITT dimensions, with an overall score of M = 4.50.

Limited Perceived Preparation (LPP) included 65 participants (15.8%) and showed consistently below-average scores across the four dimensions, with an overall mean of 3.38.

Three smaller profiles exhibited more differentiated configurations. Regulation Vulnerability (RV) included 26 teachers (6.3%) and was characterized by comparatively high perceived knowledge in Mood (M = 5.15), Psychosocial Well-being (M = 5.28), and Identity (M = 5.38), alongside markedly lower perceived knowledge in Emotional Regulation (M = 1.85). Global Vulnerability (GV) included 29 participants (7.0%) and showed the lowest scores across all dimensions, with an overall mean of 1.99. Finally, Regulation–Well-being Vulnerability (RWV) comprised 22 participants (5.3%) and showed particularly low scores in Emotional Regulation (M = 1.78) and Psychosocial Well-being (M = 2.05), while perceived knowledge related to Identity remained comparatively high (M = 5.17) (Table 4).

**Figure 1:**
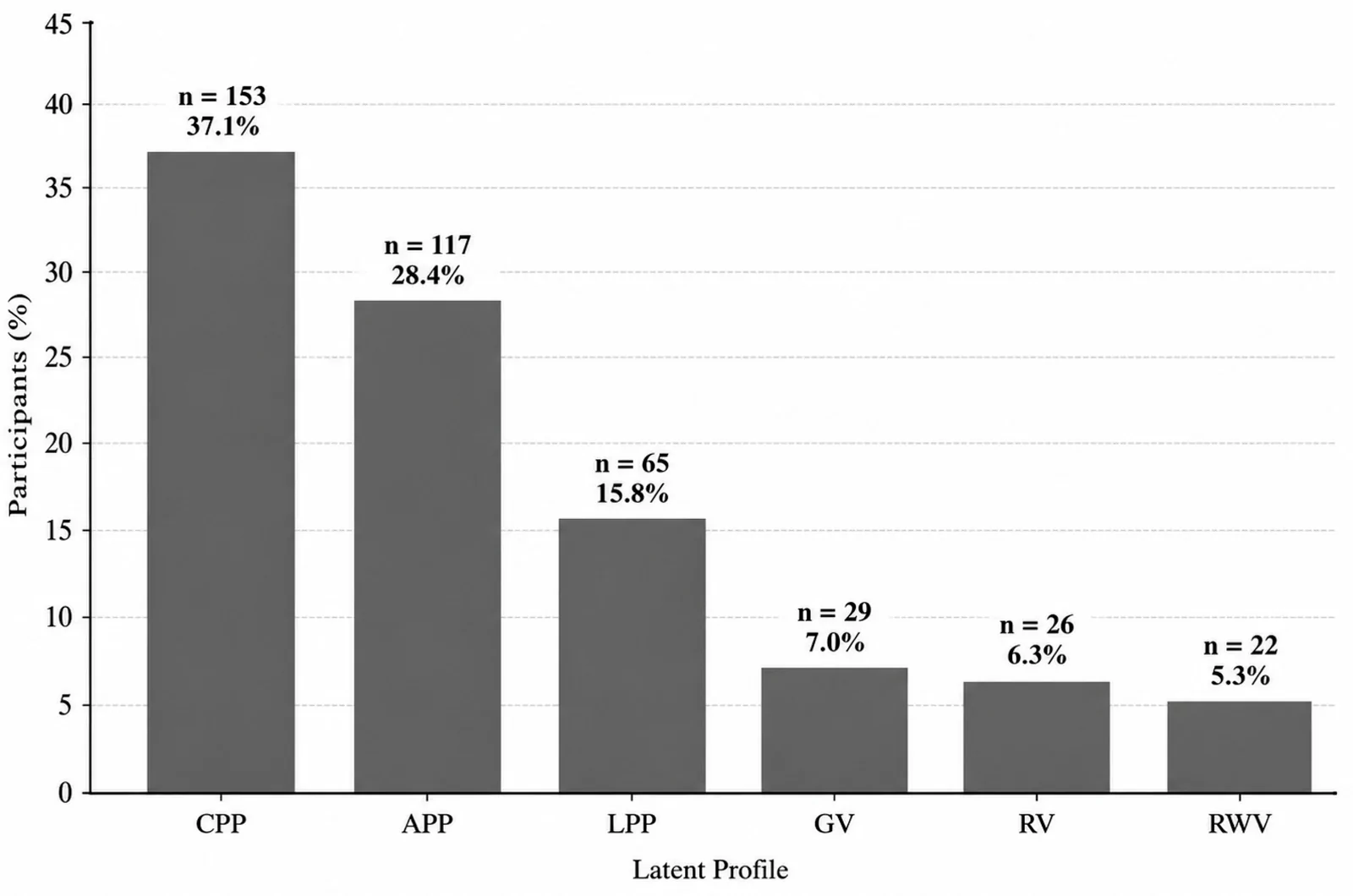
Distribution of the Six Latent Profiles of Retrospectively Perceived Knowledge Acquired in Emotional Education Among Chilean In-Service Teachers. Note: LPP = Limited Perceived Preparation; RV = Regulation Vulnerability; APP = Average Perceived Preparation; GV = Global Vulnerability; RWV = Regulation–Well-being Vulnerability; CPP = Consolidated Perceived Preparation. Bars represent the percentage of participants assigned to each profile according to their highest posterior membership probability.

The distribution of these profiles in the sample is presented in Figure 1, where the Consolidated Perceived Preparation (CPP) profile was the most prevalent (37.1%), followed by Average Perceived Preparation (APP; 28.4%) and Limited Perceived Preparation (LPP; 15.8%). The three smaller profiles were Global Vulnerability (GV; 7.0%), Regulation Vulnerability (RV; 6.3%), and Regulation–Well-being Vulnerability (RWV; 5.3%).

**Figure 2:**
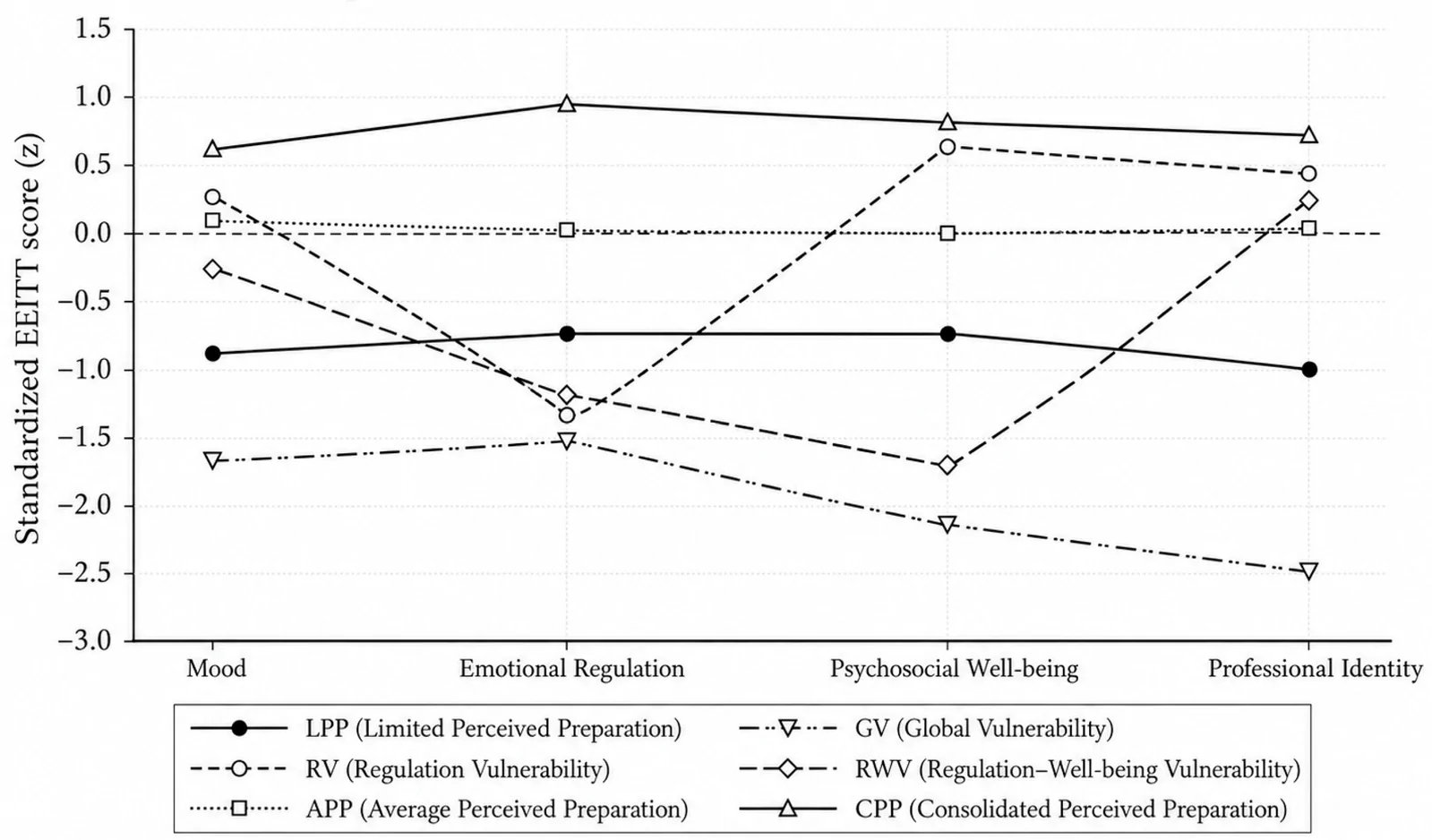
Standardized Latent Profiles of Retrospectively Perceived Knowledge Acquired in Emotional Education Across EEITT Dimensions. Note: Values represent standardized EEITT dimension scores (z-scores). Positive scores indicate values above the sample mean, whereas negative scores indicate values below the sample mean. LPP = Limited Perceived Preparation; RV = Regulation Vulnerability; APP = Average Perceived Preparation; GV = Global Vulnerability; RWV = Regulation–Well-being Vulnerability; CPP = Consolidated Perceived Preparation.

Figure 2 presents the trajectories of each profile across the four dimensions of the EEITT, revealing both quantitative and configurational differences in retrospectively perceived knowledge acquired in emotional education during initial teacher training. The Consolidated Perceived Preparation (CPP) profile showed consistently above-average scores across all four dimensions, whereas the Global Vulnerability (GV) profile showed consistently low scores, particularly in Psychosocial Well-being and Identity. The Average Perceived Preparation (APP) profile remained close to the sample mean across dimensions, while the Limited Perceived Preparation (LPP) profile showed a relatively uniform below-average configuration.

Two smaller profiles displayed particularly differentiated patterns. The Regulation Vulnerability (RV) profile showed above-average scores in Mood, Psychosocial Well-being, and Identity, but a marked reduction in Emotional Regulation. Similarly, the Regulation–Well-being Vulnerability (RWV) profile showed comparatively preserved Identity alongside substantially lower Emotional Regulation and Psychosocial Well-being. These patterns indicate that differences among the profiles involved not only overall levels of retrospectively perceived preparation but also distinct configurations across the EEITT dimensions.

### 3.3. Additional Analyses

Additional analyses were conducted to examine associations among the EEITT dimensions and to characterize the six latent profiles in relation to selected sociodemographic variables.

#### 3.3.1. Correlation Matrix

Table 5 presents the Pearson correlations among the four EEITT dimensions. All dimensions showed statistically significant positive correlations (all *p* < 0.001), with coefficients ranging from r = 0.525 to r = 0.700. The strongest correlation was observed between Psychosocial Well-being and Identity (r = 0.700), followed by Emotional Regulation and Psychosocial Well-being (r = 0.683), Emotional Regulation and Identity (r = 0.589), Mood and Identity (r = 0.569), Mood and Psychosocial Well-being (r = 0.557), and Mood and Emotional Regulation (r = 0.525).

#### 3.3.2. Sociodemographic Characterization of the Latent Profiles

To examine whether the six latent profiles differed in selected sociodemographic characteristics, age and years of teaching experience were compared across profiles. No statistically significant differences were found in age, F(5, 406) = 1.01, *p* = 0.411, η^2^ = 0.012, or teaching experience, F(5, 406) = 1.68, *p* = 0.139, η^2^ = 0.020. Both effect sizes were small. The assumption of homogeneity of variance was supported for age (Levene’s test, *p* = 0.634) and teaching experience (Levene’s test, *p* = 0.611).

Descriptively, mean age ranged from 35.8 years in the RWV profile to 40.5 years in the LPP profile, whereas mean teaching experience ranged from 8.91 years in the RWV profile to 15.0 years in the GV profile. These relatively modest differences were consistent with the non-significant omnibus tests.

Gender distribution was also examined descriptively across the six profiles. Of the total sample, 357 participants identified as female, 52 as male, one as non-binary, and two preferred not to respond. Given the extremely small frequencies in the latter two categories, several expected cell counts were insufficient for a conventional chi-square test. Therefore, no inferential comparison of gender distribution across profiles was performed.

## 4. Discussion

The research aimed to identify and characterize latent profiles of retrospectively perceived knowledge acquired in emotional education during initial teacher training in a sample of practicing Chilean teachers, using a person-centered approach. The results revealed six differentiated profiles: (a) Consolidated Perceived Preparation (CPP); (b) Average Perceived Preparation (APP); (c) Limited Perceived Preparation (LPP); (d) Regulation Vulnerability (RV); (e) Global Vulnerability (GV); and (f) Regulation–Well-being Vulnerability (RWV), which reflect heterogeneity in teachers’ retrospective perceptions of their preparation in emotional education during initial teacher training. This diversity of configurations reinforces the idea put forward by Bergman and Trost (2006) that teachers do not constitute a homogeneous group in terms of their preparation, underscoring the need to move beyond variable-centered approaches to understand the complexity of training needs (Bisquerra, 2016; Brackett et al., 2025; Corcoran & Tormey, 2013).

The identification of the Consolidated Perceived Preparation (CPP) profile as the most prevalent (37.1% of the sample) constitutes a particularly relevant finding. This profile, characterized by consistently high perceived knowledge across all dimensions (overall M = 5.43 on the 1–6 scale), suggests that more than one-third of the participating teachers retrospectively perceive their initial preparation in emotional education as comparatively strong across the dimensions assessed. At the same time, the coexistence of this profile with several profiles showing lower or more uneven patterns indicates considerable heterogeneity in how teachers recall and evaluate the emotional education received during their initial preparation. This result can be considered alongside the observations of Corcoran and Tormey (2013) and Valente et al. (2019), who warned that although teacher training curricula have begun to incorporate socio-emotional content, it is often addressed in a cross-cutting and superficial manner, without delving into specific dimensions such as emotional regulation or identity management. This pattern may be considered in relation to the curricular structure of pedagogy programs in Chile, which prioritizes disciplinary and pedagogical content over socio-emotional dimensions (Ávalos, 2014; Bächler Silva et al., 2020), combined with the absence of systematic emotional education policies that would allow for integrated competency development. Rather than indicating a uniformly insufficient preparation, the present findings suggest that perceived exposure to and consolidation of emotional education content may vary substantially across teachers. Jennings and Greenberg (2009) highlight that teacher emotional competence, understood as the effective application of emotional knowledge in practice, is a fundamental predictor of classroom climate and student well-being. However, the present findings concern perceived knowledge acquired during initial training and should not be interpreted as evidence of teachers’ current emotional competence.

A finding that deserves special attention is the presence of two profiles characterized by marked difficulties in Emotional Regulation: Regulation Vulnerability (RV; 6.3%) and Regulation–Well-being Vulnerability (RWV; 5.3%). The RV profile was especially distinctive because teachers reported comparatively high perceived knowledge in Mood (M = 5.15), Psychosocial Well-being (M = 5.28), and Identity (M = 5.38), alongside substantially lower perceived knowledge in Emotional Regulation (M = 1.85). The RWV profile showed a related but more extensive pattern, combining low Emotional Regulation (M = 1.78) and Psychosocial Well-being (M = 2.05) with comparatively preserved Identity (M = 5.17). These configurations are particularly relevant because Emotional Regulation was also the dimension with the lowest mean in the total sample (M = 3.54). Thus, the person-centered analysis extends the variable-centered descriptive result by showing that low perceived preparation in emotional regulation is not distributed uniformly across teachers but is especially pronounced within specific subgroups. This pattern is consistent with concerns raised by G. A. Fuentes-Vilugrón et al. (2021) about gaps in emotional training in Chile (Bächler Silva et al., 2020; G. Fuentes-Vilugrón et al., 2023; Riquelme et al., 2019). The findings may reflect differences in the extent to which teachers recall having received specific conceptual or practical preparation related to emotional regulation during their initial teacher training. Given the retrospective design, however, the study cannot determine whether these differences originate exclusively from initial training or whether subsequent professional experiences influence teachers’ current appraisal of that training. From a training perspective, the identification of these subgroups suggests that emotional regulation may not be perceived uniformly across teachers and that differentiated training needs should be considered. Importantly, the present findings do not establish that teachers in the RV or RWV profiles demonstrate less effective emotional regulation in professional practice.

The profiles located toward the lower end of perceived preparation also deserve attention. The Global Vulnerability (GV) profile, which represented 7.0% of the sample, showed the lowest overall level of retrospectively perceived knowledge (M = 1.99) and consistently low scores across all four dimensions. Particularly low values were observed for Emotional Regulation (M = 1.31), Psychosocial Well-being (M = 1.47), and Identity (M = 2.00). This pattern differs from the more domain-specific configurations observed in RV and RWV and suggests that a small but meaningful subgroup retrospectively perceives broadly limited preparation in emotional education during initial teacher training. Although previous literature emphasizes the importance of systematically integrating emotional education into teacher preparation (Bisquerra, 2016; Brackett et al., 2025), the present results cannot establish whether teachers in this profile currently demonstrate lower socio-emotional competence or less effective pedagogical practices. Rather, the profile identifies a subgroup reporting comparatively low perceived preparation across the content domains assessed by the EEITT.

The Limited Perceived Preparation (LPP) and Average Perceived Preparation (APP) profiles further illustrate that the identified solution cannot be reduced solely to a contrast between highly and poorly prepared teachers. LPP represented 15.8% of the sample and showed a relatively uniform below-average pattern across the four dimensions (overall M = 3.38), whereas APP represented 28.4% and remained close to the sample mean across dimensions (overall M = 4.50). Together, these two profiles accounted for almost half of the sample and occupied intermediate positions between the more clearly consolidated CPP profile and the more pronounced difficulties observed in GV, RV, and RWV. Their presence reinforces the value of a person-centered approach by identifying gradations as well as distinct configurations in teachers’ retrospective perceptions of their preparation.

The comparison between profiles reveals a consistent pattern: Emotional Regulation emerges as the dimension showing some of the most pronounced differences across configurations. It was comparatively low in LPP, markedly low in RV, GV, and RWV, approximately average in APP, and comparatively high only in CPP. Psychosocial Well-being also played a central role in differentiating RWV and GV from other profiles. In contrast, Identity was comparatively preserved in several configurations, including RV and RWV, indicating that perceived preparation across EEITT domains does not necessarily vary uniformly within individuals. These findings suggest that teachers may retrospectively perceive specific strengths and gaps within their initial preparation rather than simply reporting globally high or low levels of emotional education. The prominence of Emotional Regulation as a differentiating dimension can be considered in light of previous literature emphasizing emotional regulation as a central component of teachers’ emotional functioning and educational practice (Brackett et al., 2025; Hoffmann et al., 2020; Jennings & Greenberg, 2009). The mood and psychosocial well-being dimensions presented comparatively higher perceived knowledge in several profiles, which may reflect differences in teachers’ exposure to, recall of, or familiarity with these topics during and after their initial preparation.

The absence of statistically significant differences in age and teaching experience across the six profiles provides additional context for interpreting these patterns. Neither age (*p* = 0.411, η^2^ = 0.012) nor years of teaching experience (*p* = 0.139, η^2^ = 0.020) showed meaningful differences across profiles, suggesting that the identified configurations cannot be readily attributed to these sociodemographic characteristics. Gender distributions were examined descriptively; however, the very small number of participants in some gender categories prevented a reliable inferential comparison. Consequently, future studies with more balanced samples are needed to examine whether profile membership varies systematically according to gender or other sociodemographic and professional characteristics.

In practical terms, the findings of this study have potential implications for initial and continuing teacher training. It is important to note that these profiles reflect retrospectively perceived knowledge acquired during initial teacher training and not objectively assessed knowledge or demonstrated emotional competence in current professional practice. The differentiation of profiles suggests that teachers may perceive different training needs across the dimensions of emotional education. Teachers exhibiting patterns similar to GV or LPP may potentially benefit from professional development opportunities addressing emotional education more broadly, whereas the RV profile highlights Emotional Regulation as a particularly salient area of perceived limited preparation. For the RWV profile, both Emotional Regulation and Psychosocial Well-being appear to constitute comparatively weak areas despite relatively high perceived preparation in Identity. APP reflects a more intermediate configuration, while CPP represents comparatively high perceived preparation across the assessed dimensions. However, these patterns should not be used to prescribe interventions solely on the basis of latent profile membership. Further research using objective or observational measures would be required before considering teachers with comparatively high perceived preparation, such as those classified in CPP, as potential peer facilitators or models of effective practice. Therefore, rather than prescribing profile-specific interventions directly from the present findings, the identified profiles may serve as an empirical basis for designing and subsequently evaluating differentiated professional development strategies. Such interventions should incorporate additional assessments of teachers’ actual knowledge, skills, and classroom practices. This differentiated intervention approach responds to recommendations about the need to design training pathways tailored to the specific characteristics of teachers to maximize the efficiency of training resources (Avidov-Ungar, 2025; Frerejean et al., 2021; Ndaruhutse, 2022; Somabut et al., 2026).

### Limitations

The limitations relate to first, the use of non-probability convenience sampling, which limits the generalization of the results to the entire population of practicing Chilean teachers. Although the sample of 412 participants is adequate for latent profile analysis (Spurk et al., 2020), the overrepresentation of females (86.7%), while reflecting the reality of the feminization of the teaching profession in Chile, prevents robust differential analyses by gender and the exploration of possible profile configurations specific to male or gender-diverse teachers. This gender imbalance is particularly relevant given that women tend to report higher levels of emotional expressivity and greater use of cognitive reappraisal strategies compared to men (Gross & John, 2003), which may have influenced the self-reported scores and limits the extent to which the identified profile structure can be assumed to generalize equally across gender groups. Given the extremely small number of non-binary participants and participants who preferred not to report gender, inferential comparisons of profile membership by gender were not considered statistically appropriate.

Second, the retrospective nature of the study relies on participants’ memories of their initial training, which may be subject to recall bias or influenced by subsequent professional experiences. Although retrospective evaluations of training are common in educational research and can provide valuable insights into the perceived long-term impact of initial preparation, this does not eliminate the possibility that current professional experiences may shape participants’ recollections and evaluations of their initial teacher training.

Third, the study is based on a self-report instrument (EEITT Scale), which measures the retrospectively perceived knowledge acquired during initial teacher training, not actual knowledge or emotional competence. Self-report measures may be influenced by social desirability biases, distorted self-perception, or lack of self-awareness on the part of participants (Corcoran & Tormey, 2013). Therefore, the identified profiles should be interpreted as configurations of perceived knowledge rather than as profiles of teachers’ actual emotional competence.

Fourth, the study lacks external validation through objective measures of teacher performance or well-being that could confirm the practical significance of the identified profiles. Fifth, although some of the six profiles differed primarily in their overall level of perceived preparation, others showed more distinctive configurations across dimensions, particularly in Emotional Regulation and Psychosocial Well-being. In addition, classification uncertainty was somewhat greater for the Average Perceived Preparation profile (AvePP = 0.765), which should be considered when interpreting this profile.

Future studies could complement these measures with objective assessments of emotional competence, classroom observations, or in-depth interviews to triangulate information. Specifically, to externally validate the identified profiles, future research should examine whether teachers from different profiles exhibit distinct instructional behaviors in the classroom, correlate profile membership with objective well-being indicators (such as validated measures of burnout, job satisfaction, or emotional exhaustion), and explore through qualitative methods the professional experiences and training needs specific to each profile. The cross-sectional design of the study prevents establishing causal relationships or examining the stability of profiles over time. It is not possible to determine whether the identified profiles remain stable throughout the teaching career or whether they change as a result of work experience or training interventions (Klusmann et al., 2016). Longitudinal studies are required to understand the trajectory of these profiles and the factors that influence their evolution.

Finally, the study did not consider contextual variables that could influence the configuration of profiles, such as the type of training institution, the geographic region of training or professional practice, or the socioeconomic characteristics of the educational establishments where teachers work. In addition, information regarding teaching level, subject area, type of initial teacher education program, year of graduation, and additional training in emotional education was not collected. Given the wide age range of the sample and the retrospective nature of the EEITT, these variables may be particularly relevant, as teachers from different generations may have experienced different initial teacher education curricula and opportunities for emotional education. Incorporating these variables in future analyses could enrich the understanding of the factors associated with each profile (Assaél et al., 2018).

## 5. Conclusions

Latent profile analysis shows that the perception of knowledge in emotional education acquired during initial training is not homogeneous but rather organized into six differentiated configurations. The identification of these profiles reveals that the largest subgroup reported consistently high levels of perceived preparation across the four dimensions (CPP profile, 37.1%, M = 5.43), while the APP profile (28.4%, M = 4.50) showed a more intermediate configuration and the LPP profile (15.8%, M = 3.38) reported comparatively lower perceived preparation across dimensions. In addition, smaller subgroups showed more specific patterns of perceived training gaps, particularly in Emotional Regulation (RV: 6.3%, M = 4.41) and in Emotional Regulation and Psychosocial Well-being (RWV: 5.3%, M = 3.41), whereas the GV profile (7.0%, M = 1.99) exhibited consistently low perceived preparation across all dimensions. These findings underscore the need to abandon homogeneous training approaches and move toward differentiated interventions that recognize the diversity of teacher trajectories and perceived training needs, while recognizing that the identified profiles reflect retrospectively perceived preparation rather than objectively assessed knowledge or emotional competence. Accordingly, the profiles may provide an empirical basis for designing and evaluating differentiated professional development strategies rather than constituting direct prescriptions for intervention, positioning latent profile analysis as a useful methodological tool for designing more precise, equitable, and person-centered educational and training strategies.

## Figures and Tables

**Table 1:** Sociodemographic Descriptive Statistics and EEITT Scores.

**Variable**	**M**	**SD**	**Median**	**Range**
Age (years)	39.2	9.34	38	23–76
Years of service	12.6	8.52	11	1–45
**EEITT Factors**	**M**	**SD**	**Median**	**Range**
F1: Mood	4.91	1.05	5.16	1–6
F2: Emotional regulation	3.54	1.45	3.80	1–6
F3: Psychosocial well-being	4.40	1.39	4.83	1–6
F4: Identity	4.86	1.17	5.00	1–6
Total Scale Score	4.43	1.07	4.61	1–6
**Gender**	***n***			**Percentage**
Female	357			86.7%
Male	52			12.6%
Non-binary	1			0.2%
Prefer not to respond	2			0.5%

**Table 2:** Reliability Analysis: Cronbach’s Alpha and McDonald’s ω.

Factors	Cronbach’s Alpha	McDonald’s ω
F1: Mood	0.917	0.930
F2: Emotional regulation	0.910	0.919
F3: Psychosocial well-being	0.893	0.908
F4: Identity	0.901	0.916
Total Scale Score	0.860	0.866

**Table 3:** Model Fit Indices for Latent Profile Solutions.

Profiles	AIC	BIC	SABIC	Entropy	BLRT *p*	Smallest Profile (%)
1	4688.82	4720.98	4695.60	—	—	100.00
2	4062.49	4114.77	4073.51	0.899	0.010	27.18
3	3859.90	3932.28	3875.16	0.903	0.010	7.28
4	3787.40	3879.88	3806.90	0.804	0.010	7.04
5	3766.10	3878.68	3789.83	0.804	0.010	7.04
6	3684.71	3817.40	3712.69	0.824	0.010	5.34
7	3659.77	3812.57	3691.98	0.816	0.010	4.61
8	3668.60	3841.50	3705.06	0.740	0.921	4.61

Note. AIC = Akaike Information Criterion; BIC = Bayesian Information Criterion; SABIC = sample-size-adjusted Bayesian Information Criterion; BLRT = Bootstrap Likelihood Ratio Test. The six-profile solution was retained based on the joint consideration of model fit, classification quality, the approximately 5% profile-size benchmark, stability, parsimony, and substantive interpretability.

**Table 4:** Mean EEITT Scores and Classification Quality Across the Six Latent Profiles.

Profile	*n* (%)	Mood	Emotional Regulation	Psychosocial Well-Being	Identity	Overall M	AvePP
LPP	65 (15.8%)	3.98	2.47	3.38	3.69	3.38	0.916
RV	26 (6.3%)	5.15	1.85	5.28	5.38	4.41	0.841
APP	117 (28.4%)	5.05	3.61	4.44	4.92	4.50	0.765
GV	29 (7.0%)	3.18	1.31	1.47	2.00	1.99	0.984
RWV	22 (5.3%)	4.63	1.78	2.05	5.17	3.41	0.886
CPP	153 (37.1%)	5.54	4.91	5.55	5.71	5.43	0.914

Note. Values for the EEITT dimensions and overall score are reported on the original 1–6 scale. AvePP = average posterior probability of membership in the assigned profile. LPP = Limited Perceived Preparation; RV = Regulation Vulnerability; APP = Average Perceived Preparation; GV = Global Vulnerability; RWV = Regulation–Well-being Vulnerability; CPP = Consolidated Perceived Preparation. Higher scores indicate greater retrospectively perceived knowledge acquired in emotional education during initial teacher training.

**Table 5:** Pearson Correlations Among EEITT Dimensions.

	Mood	ER	PWB	PI
Mood	1.000	0.525	0.557	0.569
ER	0.525	1.000	0.683	0.589
PWB	0.557	0.683	1.000	0.700
PI	0.569	0.589	0.700	1.000

Note: All correlations were statistically significant at *p* < 0.001. ER = Emotional Regulation; PWB = Psychosocial Well-being.

## Data Availability

The main data supporting the results of this study are included within the article.

## Supplementary Materials

The following supporting information can be downloaded at: https://www.mdpi.com/article/10.3390/bs16091469/s1, Material S1: Complete 23-item EEITT Scale; Material S2: Complete R code for the descriptive, reliability, correlational, and latent profile analyses.
