# Supplementary material for: Latent Profiles in the Perception of Knowledge in Emotional Education During Initial Teacher Training: A Retrospective Study of Chilean In-Service Teachers
Source: Behav Sci (Basel). 2026 Aug 24;16(9):1469. doi: 10.3390/bs16091469 (PMC13603536; doi:10.3390/bs16091469)
Supplement: Supplementary file 1 [file behavsci-16-01469-s001.zip › Supplementary_Material_S1-SCALE-EEITT-SPA.pdf]

Escala de percepción del conocimiento adquirido en educación emocional durante la formación inicial docente

Gerardo Fuentes, Flavio Muñoz, Rafael Bisquerra, Enrique Riquelme, José Luis Ramos,
Felipe Caamaño, Edgardo Miranda, Carlos Arriagada, Ekaterina Legaz & Gerardo Muñoz-Troncoso

Esta escala está enfocada al análisis de la formación inicial docente respecto a las áreas de salud mental y educación emocional, consideradas como fundamentales en el proceso de enseñanza y aprendizaje. En este sentido, los participantes son estudiantes de carreras de pedagogía en universidades chilenas, y/o profesores en ejercicio. Las opciones de respuesta las conforma una escala tipo Likert de 6 puntos. La cantidad de opciones es par, para evitar la tendencia a seleccionar alternativas intermedias. Asimismo, el número de opciones para tener mayor variabilidad y la posibilidad de emplear distintos estimadores durante los análisis factoriales.

La estructura del instrumento está compuesta por 23 ítems, agrupados en 4 factores de primer orden, tal como se aprecia en la siguiente tabla. Se estimará también un modelo con un factor de segundo orden si el ajuste es igual o superior al de primer orden, con la finalidad de simplificar la medición de la influencia de algunas variables sobre el constructo.

Factores	Cantidad de ítems	Numero de los ítems
Estado de ánimo	6	1 al 6
Regulación emocional	5	7 al 11
Bienestar psicosocial	6	12 al 17
Identidad	6	18 al 23

Información demográfica

1.- Género

a) Masculino
b) Femenino
c) No binario
d) Prefiero no responder
e) Otro: ___________________

2.- Edad

____

3.- Nacionalidad[1]

Chilena
Otra: ____________

4.- ¿Pertenece a algún pueblo originario?

a) Sí
a) No
b) No lo sé

5.- ¿Señale a qué pueblo originario pertenece?

a) (Lista de pueblos originarios)
b) Otra: ______________

6.- Registre el nombre del pueblo originario al que pertenece.

____

7.- Carrera que cursa (si es estudiante) o que cursó (si es profesor)

____

8.- ¿Qué año de la carrera cursa actualmente? (pregunta para estudiantes)

____

9.- ¿Cuántos años lleva ejerciendo? (pregunta para profesores)

____

10.- Región

____

11.- Comuna

____

[1] Sobre la versión web: Quienes seleccionen nacionalidad chilena y reconozcan ascendencia indígena, serán enviados la lista sobre pueblos originarios del territorio chileno. Los participantes que registren otra nacionalidad y reconozcan ascendencia indígena, serán enviados a la opción para ingresar manualmente el nombre de la etnia.

Consentimiento informado y aviso de confidencialidad

Por favor lea esta información cuidadosamente antes de decidir si desea participar. El estudio busca conocer la percepción respecto a la formación recibida por los estudiantes de pedagogía, en las áreas de salud mental y educación emocional. Usted ha sido invitado a participar porque en la actualidad cursa alguna carrera de pedagogía. Responder el cuestionario implica que la información que se registre formará parte de una base de datos, que será empleada en futuros estudios sobre los temas referidos.

El estudio, considera todos los aspectos éticos consignados en la declaración de Helsinki de 2000 y en la Declaración de Singapur de 2010 sobre integridad en la investigación en seres humanos. En consecuencia, se realiza en conformidad a los acuerdos deontológicos internacionales y a lo referido en la Ley 20.120 vigente en Chile.

PARTICIPACIÓN: Contestar un cuestionario online de aproximadamente 9 minutos compuesto por cinco escalas: (1) Estado de ánimo, 6 ítems; (2) Regulación emocional, 5 ítems; (3) Bienestar psicosocial, 6 ítems; y (5) Identidad, 6 ítems.

SIN RIESGOS: No existe ningún riesgo anticipado asociado a participar en esta investigación, más allá de aquellos relacionados con ver una pantalla (computador, tablet, teléfono u otros aparatos digitales) durante 9 minutos y contestar el cuestionario sobre la información que se verá en pantalla.

CONFIDENCIALIDAD: Toda opinión o información que usted entregue será tratada de manera confidencial. Nunca será revelada su identidad ni detalles que permitan identificarlo. Además, no es posible que su nombre sea relacionado con sus respuestas, dado que la información se registra en formularios diferentes[2].

SUS DATOS SERÁN RESGUARDADOS en un archivo digital, al que sólo tendrán acceso mediante contraseña personal los encargados de las investigaciones que se realicen, quienes comprometen una garantía de confidencialidad.

PARTICIPACIÓN VOLUNTARIA: La participación es completamente voluntaria. Se puede retirar en el momento que estime conveniente. Para ello, basta con cerrar o abandonar la página web del cuestionario.

CONTACTO: Si usted tiene alguna consulta o preocupación respecto a sus derechos como participante, puede contactar a: Gerardo Fuentes (gerardo.fuentes@uautonoma.cl); Flavio Muñoz (flavio.munoz@uct.cl)

---

[2] Esto en la lógica que la versión web puede iniciar con una invitación, donde (de acuerdo con lo señalado por algunos revisores) pondrán su correo y/o su nombre a fin de validar que aceptan o no participar. Luego, serían redireccionados a un formulario distinto, donde se contine la escala propiamente tal.

Instrucciones para responder

A continuación, presentamos una serie de afirmaciones relacionadas con los conocimientos que usted pudo haber recibido durante su Formación Inicial Docente (FID), específicamente en torno a la salud mental y la educación socioemocional.

Indique su grado de acuerdo respecto de si en la universidad experimentó un proceso de enseñanza/aprendizaje sobre el área que aborda cada ítem. Las opciones de respuesta son seis: 1 = Muy en desacuerdo; 2 = En desacuerdo; 3 = Algo en desacuerdo; 4 = Algo de acuerdo; 5 = De acuerdo; 6 = Muy de acuerdo [3].

I.- Dimensión Estado de Ánimo

Responda considerando si los procesos de enseñanza/aprendizaje mencionados en las afirmaciones los experimentó durante la **carrera que cursa o que cursó**. Cuando un ítem lo ubique en el **rol de profesor(a)**, respóndalo pensando en su ejercicio **como futuro docente** o, si ya ejerce, en su práctica profesional actual.

1.- Aprendí que mantener estado de ánimo positivo favorece los procesos de aprendizaje.

Muy en desacuerdo	**1**	**2**	**3**	**4**	**5**	**6**	*Muy de acuerdo*

2.- Me enseñaron que la motivación es fundamental para el desarrollo de aprendizajes significativos.

Muy en desacuerdo	**1**	**2**	**3**	**4**	**5**	**6**	*Muy de acuerdo*

3.- Me enseñaron que debo transmitir un estado de ánimo positivo durante las clases.

Muy en desacuerdo	**1**	**2**	**3**	**4**	**5**	**6**	*Muy de acuerdo*

4.- Me enseñaron que mediar en la resolución de conflictos entre estudiantes, permitiría que éstos mejoren sus niveles de motivación hacia el aprendizaje.

Muy en desacuerdo	**1**	**2**	**3**	**4**	**5**	**6**	*Muy de acuerdo*

[3] La versión web dispone de todas las opciones de respuesta, en palabras, no en números como en esta versión de Word.

5.- Aprendí que la desmotivación de los estudiantes puede influir en su estado de ánimo.

Muy en desacuerdo	**1**	**2**	**3**	**4**	**5**	**6**	*Muy de acuerdo*

6.- Aprendí que un profesor(a) desanimado o con desgano podría generar ese mismo estado de ánimo en los estudiantes.

Muy en desacuerdo	**1**	**2**	**3**	**4**	**5**	**6**	*Muy de acuerdo*

II. Dimensión Regulación Emocional

Responda considerando si los procesos de enseñanza/aprendizaje mencionados en las afirmaciones los experimentó durante la **carrera que cursa o que cursó**. Cuando un ítem lo ubique en el **rol de profesor(a)**, respóndalo pensando en su ejercicio **como futuro docente** o, si ya ejerce, en su práctica profesional actual.

7.- Aprendí acerca del concepto de regulación emocional

Muy en desacuerdo	**1**	**2**	**3**	**4**	**5**	**6**	*Muy de acuerdo*

8.- Me enseñaron sobre las influencias de la regulación emocional en los procesos de enseñanza-aprendizaje.

Muy en desacuerdo	**1**	**2**	**3**	**4**	**5**	**6**	*Muy de acuerdo*

9.- Aprendí herramientas para educar las emociones de mis estudiantes para el desarrollo de su bienestar personal y social.

Muy en desacuerdo	**1**	**2**	**3**	**4**	**5**	**6**	*Muy de acuerdo*

10.- Me enseñaron que profesores(as) regulados emocionalmente se traduce en estudiantes regulados emocionalmente.

Muy en desacuerdo	**1**	**2**	**3**	**4**	**5**	**6**	*Muy de acuerdo*

11.- Me enseñaron que debo dominar los documentos normativos asociados a la educación socioemocional en contextos educativos.

Muy en desacuerdo	**1**	**2**	**3**	**4**	**5**	**6**	*Muy de acuerdo*

III. Dimensión Bienestar Psicosocial

Responda considerando si los procesos de enseñanza/aprendizaje mencionados en las afirmaciones los experimentó durante la **carrera que cursa o que cursó**. Cuando un ítem lo ubique en el **rol de profesor(a)**, respóndalo pensando en su ejercicio **como futuro docente** o, si ya ejerce, en su práctica profesional actual.

12.- Aprendí acerca de los conceptos de autoestima y/o autoimagen.

Muy en desacuerdo	**1**	**2**	**3**	**4**	**5**	**6**	*Muy de acuerdo*

13.- Me enseñaron que el autoconcepto y autoimagen, es un componente que influye en el desarrollo de aprendizaje de los estudiantes.

Muy en desacuerdo	**1**	**2**	**3**	**4**	**5**	**6**	*Muy de acuerdo*

14.- Aprendí que un adecuado autoconcepto proyecta mayor confianza y seguridad en los estudiantes favoreciendo su salud mental.

Muy en desacuerdo	**1**	**2**	**3**	**4**	**5**	**6**	*Muy de acuerdo*

15.- Aprendí que el autoconcepto influye en la calidad de las relaciones sociales que se dan en el espacio escolar.

Muy en desacuerdo	**1**	**2**	**3**	**4**	**5**	**6**	*Muy de acuerdo*

16.- Me enseñaron que el autoconcepto tiene un papel fundamental en la regulación de las estrategias cognitivo-motivacionales implicadas en el aprendizaje y rendimiento académico.

Muy en desacuerdo	**1**	**2**	**3**	**4**	**5**	**6**	*Muy de acuerdo*

17.- Me enseñaron que el autoconcepto se construye basado en las relaciones que tenemos con los otros(as) y con la sociedad.

Muy en desacuerdo	**1**	**2**	**3**	**4**	**5**	**6**	*Muy de acuerdo*

IV. Dimensión Identidad

Responda considerando si los procesos de enseñanza/aprendizaje mencionados en las afirmaciones los experimentó durante la **carrera que cursa o que cursó**. Cuando un ítem lo ubique en el **rol de profesor(a)**, respóndalo pensando en su ejercicio **como futuro docente** o, si ya ejerce, en su práctica profesional actual.

18.- Me enseñaron que debo crear instancias donde mis estudiantes se sientan vistos(as) y escuchados(as).

Muy en desacuerdo	**1**	**2**	**3**	**4**	**5**	**6**	*Muy de acuerdo*

19.- Aprendí que debo crear ambientes de diálogo que contribuyan a la disminución de actitudes de discriminación en las aulas escolares.

Muy en desacuerdo	**1**	**2**	**3**	**4**	**5**	**6**	*Muy de acuerdo*

20.- Me enseñaron que los contextos escolares se caracterizan por la existencia de diversas identidades personales y sociales.

Muy en desacuerdo	**1**	**2**	**3**	**4**	**5**	**6**	*Muy de acuerdo*

21.- Aprendí que debo estar al tanto y comprender las realidades de los estudiantes para conocer sus características identitarias.

Muy en desacuerdo	**1**	**2**	**3**	**4**	**5**	**6**	*Muy de acuerdo*

22.- Me enseñaron que debo considerar las características identitarias de los estudiantes para planificar y desarrollar mis clases.

Muy en desacuerdo	**1**	**2**	**3**	**4**	**5**	**6**	*Muy de acuerdo*

23.- Aprendí que debo promover instancias para el desarrollo de la identidad de cada uno de mis estudiantes.

Muy en desacuerdo	**1**	**2**	**3**	**4**	**5**	**6**	*Muy de acuerdo*

Muchas gracias por su participación.
